# Characteristics of mentally ill offenders from 100 psychiatric court reports

**DOI:** 10.1186/1744-859X-9-4

**Published:** 2010-01-14

**Authors:** Yasser A Elsayed, Mohamed Al-Zahrani, Mahmoud M Rashad

**Affiliations:** 1 Institute of Psychiatry, Ain Shams University World Health Organization Collaborative Centre for Training and Research, Abbasia, Cairo, Egypt; 2 Al-Amal Complex for Mental Health, Dammam, Saudi Arabia; 3 Department of Psychology, South Valley University, Kena, Egypt

## Abstract

**Background:**

There is an increasing probability that the psychiatrist will, willingly or not, come into contact with mentally ill offenders in the course of their practice. There are increasing rates of violence, substance abuse and other psychiatric disorders that are of legal importance. Therefore, the aim of this work was to investigate the rates of different mental disorders in 100 court reports and to investigate the characteristics of mentally ill offenders.

**Methods:**

All cases referred from different departments of the legal system to the forensic committee for assessment of legal accountability over 13-months duration were included. A specially designed form was prepared for data collection. Cases were classified into five groups: murder, robbery, financial offences, violent and simple offences and a group for other offences. Data were subjected to statistical analysis and comparisons between different groups of subjects were performed by analysis of variance (ANOVA).

**Results:**

Men constituted 93% of cases. In all, 73% of offenders were younger than 40 years old. Schizophrenia cases made up 13% of the total, substance related cases constituted 56% and amphetamine cases alone made up 21%; 10% of cases were antisocial personality disorders, and 51% of cases were classified as having a low education level. Unemployment was found in 34% of cases. The final decision of the forensic committee was full responsibility in 46% of cases and partial responsibility in 11% of cases, with 33% considered non-responsible. A total of 58% of cases had had contact with psychiatric healthcare prior to the offence and in 9% of cases contact had been in the previous 12 weeks. A history of similar offences was found in 32% of cases. In all, 14% of the offences were murders, 8% were sexual crimes, and 31% were violent/simple crimes.

**Conclusions:**

The ability of the legal system to detect cases was good, while the ability of the healthcare system to predict crimes and offences was weak, as 58% of cases had had previous contact with the healthcare system previously. Substance abuse, especially amphetamine abuse, played an important role.

## Background

For many reasons, there is an increasing probability that the psychiatrists will, willingly or not, come into contact with mentally ill offenders in the course of their practice. There are increasing rates of violence, substance abuse and other psychiatric disorders that are of legal importance. Consequently, Western society felt a need to regulate and answer the question of what deviant mental states are of relevance to the court [1]. Although Arab countries were among the first in the world to establish mental health hospitals (in Baghdad in the year 705 AD, Cairo in 800 and in Damascus in 1270 [2]), currently most Arab countries have no mental health acts [3], no certified training in forensic psychiatry, there is little research if any in forensic psychiatry and forensic psychiatric services are poorly organised [3,4]. However, the growth in the economy of the Arab gulf countries in the last few decades has been associated with growth of all the systems needed to support this economy, including mental health and judicial systems. Motivated by the above issues this study was performed to: (1) investigate the rates of different mental disorders in 100 psychiatric court reports, (2) identify the characteristics of mentally ill offenders, and (3) revise the importance of psychiatric court reports and the obstacles that face psychiatric teams during and after assessment of subjects. Other points were also addressed, such as the ability of the legal professionals to detect mental illness during their routine work, the relation of substance abuse to crimes, the types of crimes commonly committed by mentally ill people and the ability of the mental health system to predict dangerousness of the patients. Finally, this study was a trial to review the current situation of forensic psychiatric services in the Eastern province of Saudi Arabia.

## Methods

### Location

This study was performed at the Al-Amal Complex for Mental Health, which is located in Al-Dammam, Saudi Arabia. The complex is run by the Ministry of Health of the Kingdom of Saudi Arabia (KSA). The complex has 500 beds, of which 200 are for addiction treatment, 140 for psychiatry and 160 are 'halfway house' beds. The hospital is recognised for teaching by the Saudi Council for Health Specialties and takes medical students from King Faisal University, to which the hospital is affiliated. There are many subspecialty services such as: an addiction treatment team and program, a child psychiatry team and program, a liaison psychiatry team, a community psychiatry team and a forensic psychiatry committee. The complex serves all the Eastern and Northern provinces of KSA in addition to other nearby gulf countries such as Bahrain, Kuwait, Doha, and so on. The complex receives all cases referred for assessment from the police, prisons and courts in the nearby area. The forensic psychiatry committee is one of the most important units inside the complex. The committee has a dual obligation: to the patient and to the referring agency. Among the reasons for referral to this committee are forensic problems, cases in need of a legal guardian, mental fitness to work, and so on. There are about 1,000 cases received for assessment from different sources annually. The committee is made up of a multidisciplinary team including psychiatrists, psychologists, social workers and nurses. All the investigators have been members of the team for many years. The committee holds two open sessions per week to meet patients and representatives from referring associations.

This study was approved by the scientific and ethical committee of Al-Amal Complex for Mental Health and informed consent was given by all subjects.

### Selection of the sample

All cases referred from different departments of the legal system (police, prisons, courts, and so on) to the forensic committee for assessment of criminal responsibility over 13-months duration were included. All information was collected from the patients themselves, psychiatric files and data referred from the police or the courts.

### Data collection

A specially designed form was prepared for data collection, and included demographic characteristics, clinical assessment, past history of psychiatric disorders, substance abuse and similar offences, diagnosis, dates of first contact with heath care system and legal system, source of referral, details of the case in question and the results of investigations and mental state at time of the offence. Subjects who had received less than 9 years of education were regarded as having a low education level, those who had received 9 to 12 years of education were regarded as having an intermediate level, and subjects who had received 12 years or more of education were regarded as having a high education level. All diagnoses were made according to the mini international neuropsychiatric interview (MINI), which is a short structured diagnostic interview. The scale had been translated into Arabic and validated previously [5]. However, diagnosis of organic mental disorders and personality disorders were based on the International Classification of Disease, 10th revision (ICD-10) diagnostic criteria [6] and were validated by two other consultant psychiatrists with good inter-rater reliability. Each subject was interviewed by the investigators at least once, and some patients needed more sessions to finalise their assessment.

### Statistical analysis

The main findings are presented as proportions with 95% confidence intervals (CIs). For some analytical statistics, cases were classified into five groups: murder (including murder and manslaughter), robbery (included robbery and forced robbery), financial crimes (such as loan sharking, debt, bribery and embezzlement), violent and simple offences, and a group for other offences. Comparisons between different groups of subjects were performed by analysis of variance (ANOVA) with statistical significance set at *P *< 0.05.

## Results

In this study, 93% of cases were men and only 7% were females. The mean age for the cases was 31.33 ± 4.25 years and the age range was 18 to 86 years. In all, 73% of cases were younger than 40 years old and 10% less than 20 years old. The mean ages of financial and murder groups were significantly more than other groups. A total of 51% of cases had a low education level and only 11% had a high education level. The mean for years of education was significantly lower in the robbery group (3 ± 2.7 years) than in other groups. A total of 64% of the subjects were single. Significantly, most of the financial group subjects were married and most of the violent and simple offences group were single. This was statistically significant in comparison to other groups. Unemployment was found in 34% of cases. There were significant differences between groups in employment as most of the subjects in robbery group were not employed and most of the subjects in the financial group were employed. A total of 58% of offenders had a history of previous contact with the psychiatric healthcare system prior to the offence, and significantly the mean duration of last contact with psychiatric healthcare services was less in the murder (9.5 ± 2.3 months) and robbery (10.5 ± 5.2 months) groups than in the other groups (Table 1).

**Table 1:** Demographic factors

	Total, N = 100	Murder, N = 14	Robbery, N = 16	Financial, N = 12	Violent and simple offences, N = 31	Other, N = 27	*P *value
Mean (SD) age, years	31.33 ± 4.25	40.28 ± 2.21	32.81 ± 3.32	41.43 ± 2.38	20.21 ± 3.58*	28.23 ± 3.45	0.05

Mean (SD) years of education	7 ± 2.1	8 ± 1.9	3 ± 2.7	6 ± 3.1	9 ± 3.2	10 ± 2.2	0.001

Single, n (%)	64	8 (57.1%)	11 (68.7%)	3 (25%)	24 (77.4%)	18 (66.6%)	0.01

Unemployed, n (%)	34	8 (57.1%)	15 (93.7%)	1 (8.4%)	6 (19.4%)	4 (14.9%)	0.01

Last contact in month, mean (SD)	18.7 ± 5.2	9.5 ± 2.3	10.5 ± 5.2	22.2 ± 3.9	20.2 ± 4.5	13.4 ± 4.6	0.05

Similar offences, n (%)	32	0	12 (75%)	9 (75%)	10 (32.2%)	1 (3.7%)	0.01

*P *value is significant at ≤ 0.05.

The last contact with the psychiatric healthcare system was within 3 months in 9% of cases, all of them substance abuse, within 1 year in 22% of cases, most of them substance abuse, and more than 1 year in 27% of cases. A history of similar offences was found in 32% of cases. Similar offences were significantly more common in the robbery and financial groups than in other groups. In 10% of cases referral was requested by the offenders themselves, and in 8% of cases referral was requested by a family member or a lawyer; 82% of cases were referred after an observation from the legal system (police or prison system) (Table 2).

**Table 2:** Source of referral

Referral request origin	Referred	Mentally ill
Patient	10	9

Family member or lawyer	8	8

Court	22	21

Police	42	38

Prison service	18	14

The current diagnoses of the offenders are listed in Table 3. Some of the offenders had more than one diagnosis. The most common diagnosis was substance abuse or dependence (56% of the sample). In all, 10% of the sample had no mental disorders.

**Table 3:** Current diagnoses according to mini international neuropsychiatric interview (MINI) or International Classification of Diseases, 10th revision (ICD-10) research diagnostic criteria

Diagnosis	Patients, n	Number of cases and percentage from the subgroup					*P *value
			
		Murder	Robbery	Financial	Violent and simple offences	Other	
Schizophrenia	13	3 (21.4%)	0	2 (16.6%)	3 (9.6%)	5 (18.5)	0.05

Drug abuse	56	7 (50%)	13 (81.2%)	3 (25%)	18 (58%)	15 (55.5%)	0.001

Bipolar disorder	8	0	1 (6.2%)	1 (8.3%)	1 (3.2%)	5 (18.5%)	0.04

Antisocial disorder	10	0	7 (43.7%)	0	2 (6.45%)	1 (3.7%)	0.001

Organic disorder	6	0	0	1 (8.3%)	3 (9.65%)	2 (6.15%)	0.04

Delusional disorder	8	6 (42.8%)	0	0	2 (6.45%)	0	0.001

Adjustment disorder	9	0	0	7 (58.3%)	1 (3.2%)	1 (3.7%)	0.01

Dissociative disorder	2	0	0	1 (8.3%)	1 (3.2%)	0	0.23

Schizoaffective disorder	3	0	0	1 (8.3%)	2 (6.45%)	0	0.11

Mental retardation	9	1 (7.1%)	0	0	4 (12.9%)	4 (14.8%)	0.13

No mental disorder	10	1 (7.1%)	3 (18.7%)	4 (33.3%)	2 (6.45%)	0	0.05

Comorbidity	35	4 (28.7%)	8 (50%)	8 (66.6%)	8 (25.8%)	7 (25.9%)	0.04

*P *value is significant at ≤ 0.05.

The rates of delusional disorder and schizophrenia diagnoses between murderers were significantly higher than in other groups. Also, the diagnosis of antisocial disorder was more common in the robbery group whereas diagnosis of adjustment disorder was more common in the financial group. Comorbidity was found in 35% of cases and distributed across all groups; the most common comorbidity was substance abuse, especially amphetamine abuse. Most cases with no mental disorders were in the robbery and financial groups. Persons diagnosed as having other personality disorders made up 2% of the total number of cases, major depression 4%, dysthymic disorder 1%, acute psychosis 1%, paraphilias 3%, dementia 1%, and other mental disorders 14%. Among the murder group (n = 14), four cases were amphetamine addicts, three were addicts of amphetamine plus other substances, four of them had previously been admitted to addiction units for treatment and two of them had been discharged within 2 months from the crime of murder. Out of 14 cases of murder, 8 cases had committed the crime as a result of delusions. Of 12 financial offences, 10 were failure to pay a debt or loan. One case was bribery and the final case was embezzlement. Seven cases among the cases of robbery were forced robbery. Violent and simple offences included 19 cases of physical fights, 3 of disobedience of parents, 3 cases of intruding onto others' property and 6 were minor traffic accidents.

### Other offences

Other offences (n = 27) included 8 cases of sexual crimes; 7 of the perpetrators were single, 6 had a low education level and 7 were unemployed. Two cases were homosexual acts, three were cases of paedophilia, two were cases of rape and one was a case of sexual molestation. The decision of the committee was full responsibility in four cases and partial responsibility in two cases (diagnosed as mild mental retardation with IQs 69 and 65, respectively) and no responsibility in two cases (a schizophrenic patient and a patient with organic psychosis).

Other offences included five cases of dealing in illegal narcotics. All of these cases involved substance users. One of them had an additional comorbid diagnosis of organic psychosis due to a car accident that had occurred after the crime, and he was ruled as being unfit to plead while the others were considered fully responsible.

Another five cases were accused of arson. Two of them were substance abusers and considered responsible, two were mentally retarded and considered not responsible and the last one was diagnosed as having impulse control disorder and was considered partially responsible.

There were three cases of failed suicide attempt. All were men; one was schizophrenic whereas the second was acutely psychotic and the last was dysthymic. All were considered not responsible.

Four cases were accused of various security issues: one of them took part in a terrorist attack in the country, and was diagnosed as having mild mental retardation and was not considered responsible. Two cases were trying to cross international borders; no mental disorders were found in either of them and both were considered responsible. The fourth was found to belong to a cult group, and was diagnosed as having adjustment disorder and was considered partially responsible.

Of the last two cases, one of them was referred for assessment of his mental ability to take decisions after he divorced his wife and asked to return to her again; he was found to be responsible and has no mental illness. The last case was presented to assess his mental ability to sell and buy and to sign contracts after he sold his building; he showed no mental illness and was given full responsibility for decision making.

### Decisions of the Committee

The final decisions of the committee are listed in Table 4. A decision of full responsibility was given to 46% of the offenders, 11% of offenders were considered partially responsible, 3% were unfit to plead and 7% were referred to another forensic committee. In all, 33% of cases were considered to be entirely non-responsible. All robbery and financial group cases were responsible, while most murderers were significantly not responsible in comparison to other groups. In 13% of the cases the court asked for extra details and in one case the staff of the committee were presented to the court and to the relatives of the murdered person to discuss the reasons for the decision of non-responsibility of the murderer with them.

**Table 4:** Decisions of the committee.

Decision	Total	Number (% from subgroup)					*P *value
			
		Murder	Robbery	Financial	Violent and simple offences	Other	
Responsible	46	3 (21.4%)	16 (100%)	12 (100%)	14 (45.1%)	1 (3.7%)	0.01

Irresponsible	33	11 (78.5%)	0	0	17 (54.8%)	5 (18.5%)	0.01

*P *value is significant at ≤ 0.05.

As shown in Table 5, the most common substances abused were amphetamine in 21% of cases, amphetamine with other substances in 18% of cases, alcohol in 9% of cases, khat in 4% of cases, and cannabis in 4%.

**Table 5:** Common substances abused by mentally ill offenders

Substance	N (%)
Amphetamine	21

Amphetamine with other substances	18

Alcohol	9

Khat	4

Cannabis	4

## Discussion

Mentally ill offenders present complex challenges to public policy and the criminal justice system. Their identification, assessment, processing and treatment are considered the responsibility of forensic psychiatric services in collaboration with the justice system and other legal agencies. All laws in Saudi Arabia are derived from Islamic Shariah law. Islamic philosophy acknowledges that criminal responsibility may be affected by the presence of mental illness [4]. The Prophet Mohammed is said to have regarded the insane as free of guilt for acts they may commit. Islamic law protects mentally incompetent individuals from being regarded as responsible for their crimes, but does not delineate exactly what is meant by mental incompetence, each case being left to the court to decide. The courts in Saudi Arabia are operated by religious men (Shiekh) who decide on recommendations from the forensic committee. Islamic Shariah law has a wider definition of criminal acts than in the West. Behaviours such as suicide, extramarital sexual relationship, homosexuality and alcoholism are all regarded as criminal under Islamic law.

The current study was performed to describe the process of assessment of subjects under review and to identify their characteristics, to enable the service planners to better organise and coordinate the efforts of different system partners.

In this study only 10% of cases were less than 20 years old, while other studies denoted higher percentages in younger age groups [7,8] but all their subjects were prisoners. The current study denoted that marriage and education are protective against crimes even in mentally disordered patients, as a large percentage of the offenders were single (64%) and unemployed (34%). The same finding was noticed by other studies [9]. However, this finding was noticed in mentally ill offenders as well as in offenders without psychiatric disorders, which may be attributed to increased unemployment rates and delayed marital age in this region [10,11]. Similar to other studies [10], most robbery group subjects were unemployed and their educational levels were less than other groups. Most subjects in the financial group were employed and married, which is unsurprising as financial needs increase with marriage.

Consistent with other studies, most cases in the current study were males [8,12] and previous contact with psychiatric services was found in 58% of cases [13,14]. However, the current study showed that prediction of danger in the murder and robbery groups is questionable, as the last contact of both groups with psychiatric services was significantly shorter than other groups. Skeem *et al*. [15] showed that advances in risk assessment have improved the ability to identify psychiatric patients at high risk for violence, but this was based on a well developed system of mental healthcare in the USA where training in this particular area is much better than in the Middle East. Also, the results of the current study reflect the increased rates of substance abuse in the murder and robbery groups, and ensure the importance of the presence of clear mental health acts to regulate voluntary and involuntary admissions.

Moreover, the current study found that most similar offences occurred in the robbery and financial groups and that 32% of cases had a history of similar offences before the crime under assessment, which was lower than in the study by Fulwiler *et al*. [16] in which the rate of similar offence was 68%. This may be explained by increased diagnosis of antisocial personality disorder in the robbery group and the increased levels of debt and taking of loans in the region due to the current economic crisis.

Furthermore, only 10% of cases in the current study had no mental disorders and their referral was not indicated. But this percentage is not high in comparison to other studies [17]. Also, 40% of these non-indicated cases (4 cases) were referred from prison. It seems that prisoners commonly try to feign psychiatric symptoms to gain referral for psychiatric help, which is a well known fact denoted by other studies [18]. Other referrals were almost all appropriate (Table 2), which indicates the high ability of the legal system to detect genuine disturbed behaviour.

The relationship between substance abuse and crime has been well known for some time, but according to the current study the depth of this relationship is alarming, and serves to justify a sense of urgency for intervention as the rate of substance abuse was 56%. This percentage is nearly the same as those found in New York and Washington in the USA [19]. The rate of substance abuse was much higher in an Iranian study [8] that found 73% of offenders in the prison have a lifetime history of opiate abuse; however, there are clear methodological differences between the current study and the Iranian study. In contrast, other studies [20,21] found lower rates of substance abuse and dependence (18% and 17%) than the current study. However the subjects in the former study were murderers only, and the second was a Swedish study; the rate of crime has increased greatly in Sweden in the last 10 years [9]. The high rate of substance abuse in the current study is in line with the higher rate of substance abuse in the gulf region [22,23]. Cases with dual diagnosis made up 39% of the total, and they were considered a high-risk group to commit crimes as observed by other studies [24,25]. A high rate of amphetamine abuse was found among offenders (21%) and this is due to the wide prevalence of several types of cheap amphetamine in this region [26]. The implications of these figures are significant as effective substance control might significantly influence crime rates.

Delusions were the most frequent trigger for murder, as happened in 8 cases, and delusional disorder was the most common diagnosis among murderers (6 of 14 cases) followed by schizophrenia (3 of 14). This result is logical as patients with delusional disorder are more dangerous than schizophrenic patients, as they are more likely to plan the crime. However, in their study Shaw *et al*. [20] reported that 34% of homicide offenders were mentally ill and only 5% of them were schizophrenic, but this was in a larger scale study. Additionally, 50% of murderers had a history of substance abuse, especially amphetamine, which is often adulterated with other toxic substances and one dose may lead to induced psychosis or relapse of stabilised psychotic patients, as found in another Saudi study [26].

### The decisions of the forensic committee

Full responsibility is a very difficult decision for any forensic committee as it means there is no right for the person/patient for excuse or mitigation. In KSA, if the investigation confirms full responsibility some punishments are irreversible, such as cutting the hands off for robbery, execution for murderers or even corporal punishment such as beatings or lashes. This is why a responsibility decision was not given until the completion of full data collection from all possible sources and after assessment by all means. Consequently, these patients took more time to assess and had sessions more frequently than others. The rate of full responsibility was 46%, as there was high rate of substance abuse diagnosis (56%). The rule of the committee for offenders with only a substance abuse diagnosis is full responsibility provided the person intended to take the substance and knew its prohibited nature.

Partial responsibility was given in only 11 cases. Usually this decision results from long debate, because in these cases although the persons have psychiatric diagnoses they can still realise that their behaviours are wrong but cannot control themselves adequately.

The concept of fitness to plead is firmly rooted in the soil of legal tradition. It is meant to protect the mentally ill from the rigours of the court, but if it is applied to the wrong individuals in the wrong circumstances it will stigmatise the whole process of legal accountability; hence its application is very limited in many countries, as denoted in other studies [27,28]. In the current study this decision was given in only three cases: the first case was a drug dealer who developed organic psychosis after a car accident following his crime. The second was a case of severe hebephrenic schizophrenia with marked impairment of cognitive functions, and the third was a patient with Alzheimer disease.

In contrast, the decision of referral to another committee was given when data were very poor or controversial or when the patient needed a very long period of observation. All such cases were referred to Al-Taif Mental Hospital, where the central forensic committee for Saudi Arabia is located.

### Issues and limitations

Working in forensic psychiatry is an extremely difficult job because most patients deny or exaggerate their symptoms, sources of data are questionable and cases usually present too late after the offence. Interviews with such cases can take a long time, and diagnosis may take frequent visits and may not be achieved. The absence of a mental health act, in addition to unclear regulation of the judiciary system, can cause problems during the processing of court reports. The same issue applies to lawyers, and to what extent they can interfere with the psychiatric interview and if they have the right to attend this interview or not. Staff working on the forensic committee receive no monetary benefits and are burdened with other duties. Consequently, psychiatrists usually prefer not to work on such forensic committees.

The scope of this study was wide, as it included all types of offences and all psychiatric disorders, so it was difficult to include more analytical statistics and correlations between different groups of disorders and offences in addition to the fact that the number of subjects in each subgroup was small. This study should be followed by in-depth analytical studies to investigate the different factors influencing different psychiatric disorders and their relationships to different offences.

## Conclusions and implications

Proper court reporting is important to know who is responsible and who is not responsible in the justice system. It is essential to prevent escape from justice through psychiatric defences, and to prevent further crimes if possible. The need for a mental health act is important to define the responsibilities and extent of authority of professionals and institutions, and to prevent the abuse of mentally ill patients by families, professionals and the legal system. Primary prevention, treatment and rehabilitation of patients with substance abuse and dependence have a strong relationship with crime rates, and should be a focus of attention for service planners. The role of the psychiatrist as an expert witness in the court is still weak, and in need of further delineation. The training of mental health professionals in this key area of psychiatry is also weak and in need of strong support. Finally, court reporting is a highly professional job and a piece of 'psychiatric art'. It is the conclusion of long periods of assessment, investigation and discussion, and can impact the life of many persons negatively or positively; consequently, mental health professionals should approach it with a desire to complete it adequately and perfectly.

## Competing interests

The authors declare that they have no competing interests.

## Authors' contributions

All authors conceived of the study and participated in its design and coordination. YAE administered the instrument and collected the data. MA-Z directed and oversaw the statistical analysis. MMR participated in data collection and conducted statistical analysis. All authors participated in the writing and revision and approved the final manuscript.
